# Public health programmes for vitamin A deficiency control

**Published:** 2013

**Authors:** Maaike Bruins, Klaus Kraemer

**Affiliations:** Nutrition Scientist: Sight and Life, Basel, Switzerland. maaike.bruins@sightandlife.org; Director: Sight and Life, Basel, Switzerland, Adjunct Associate Professor: Johns Hopkins Bloomberg School of Public Health, Baltimore, USA.

**Figure:**
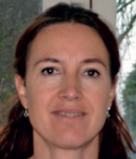
Maaike Bruins

**Figure:**
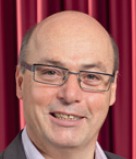
Klaus Kraemer

Since the early 1980s, when it was first realised that children with the eye signs of vitamin A deficiency (VAD) had a higher mortality rate than children in the same communities who did not have these signs, there have been many large-scale, community-based trials to assess whether improving the vitamin A status of young children improves child health and survival. Many of the trials involved intermittent supplementation with high-dose vitamin A, but food-based interventions such as food fortification have also been assessed.

Findings from the large number of randomised controlled trials of supplementation of children aged 6–59 months have been pooled, showing that intermittent supplementation with high-dose vitamin A has a major impact on child mortality in communities of children at risk of VAD. When all the results are combined, the mortality rate of the children given supplements was 24% lower than that of the children not given supplements. Some of these trials also showed a reduction in diarrhoea, measles, night blindness and other signs of xerophthalmia. (The results of trials of children under the age of 6 months, and of newborn infants and mothers, are less clear). Analysis of randomised controlled trials of vitamin A-fortified foods showed significant impacts on serum retinol concentration and haemoglobin levels.^1^

The results of all these trials have led to global initiatives (see pages 61 and 62) to control VAD in children. There has been progress in many countries, but further action is needed to increase the number of countries implementing programmes to address VAD.

## Food-based strategies

Food-based strategies are a long-term approach to controlling VAD.

### Fortification of staple foods

In some countries, where industrial and commercial infrastructure is adequate, fortification of food staples like flour, sugar, oil or condiments with pre-formed vitamin A (retinol) can be a very cost-effective way of reducing VAD. To be successful, the fortified food must be eaten by those at risk of VAD (young children and mothers) on a regular basis. To increase acceptability, the appearance, shelf-life and costs of the fortified and non-fortified food should be comparable. Fortification programmes demonstrate that with high coverage and adequate fortificant levels, food fortification can improve vitamin A status, and thus have a positive health impact.

### Multi-micronutrient powders

Home fortification with multi-micronutrient powders (MNPs) has been successfully used in some countries and is being adopted by others. Mothers are taught how to add sachets of micronutrient powder to their child's food and how often this should be done, depending on the nutritional value of the local staple used to prepare the food (e.g. maize or rice porridge). Additional information on hygiene, health, nutrition, and child development is provided as an integral part of this approach and, depending on the programme, the micronutrient powders can be purchased or are distributed for free. Home fortification has been used with success in refugee camps, emergency situations, in child health and nutrition programmes, and in school feeding programmes.^2^ It is foreseen that, in coming years, large-scale interventions will be initiated to reach even more children and other target groups, including children and adolescents in schools.

### Selective breeding and biofortification

Increasing intakes of vitamin A through selective breeding and biofortification of staples, such as orange-fleshed sweet potatoes or orange maize, can be another approach. Compared with food fortification, however, biofortification may not be as effective. The form of vitamin A used in food fortification is more effective than plant sources at improving vitamin A status. Health education might also be required to reassure mothers that the more orange foods are healthy and not harmful.

**Figure:**
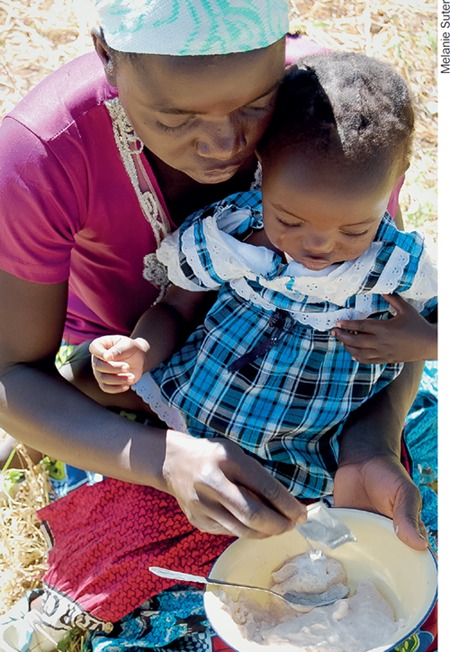
A mother in Zambia adds multi-micronutrient powder to her daughter's porridge

### Dietary diversification and improvement

Dietary diversification and improvement, including ensuring regular access to foods that are naturally rich in vitamin A, is also important in the long run.

For example, some countries are emphasising feeding programmes for preschool-aged children. Encouraging exclusive breastfeeding is another important strategy, as breast milk is a very important source of vitamin A. Breastfeeding is an important means of reducing VAD among infants and young children.

Showing people how to grow plants rich in vitamin A throughout the year, and how to store and cook them, is the most sustainable long-term food-based approach.

Even in areas where water and land are scarce, using innovative approaches to home gardening can give adequate yields for a family. If a family can also keep chickens then this improves their protein intake, and egg yolk is also an excellent source of vitamin A.

## Vitamin A supplementation

Vitamin A supplementation (page 71), when implemented on a very large scale, is a fast and cost-effective intervention to improve the vitamin A status of populations.

Vitamin A supplementation guidelines for the prevention of VAD recommend that high-dose supplements should be given to children aged 6–59 months in settings where VAD is a public health problem. In areas where VAD is a severe public health problem, low-dose vitamin A supplements are also recommended for pregnant women.

The 2011 World Health Organization (WHO) guidelines on vitamin A supplementation focus on preventive supplementation. They also contain guidelines for treatment of clinical cases of xerophthalmia and measles, and information about repeated high-dose vitamin A supplementation.

The best way of increasing coverage is to make sure that vitamin A supplementation is an integral part of child health services. For example, The Integrated Management of Childhood Illness programme which is used as the basis for services for under 5-year-olds in many countries in Africa, emphasises vitamin A supplementation. Coverage can also be increased by including supplementation during national immunisation campaigns.

## Impact of VAD control programmes

Vitamin A supplementation is very cost-effective. Capsules cost just a few cents and the potential of vitamin A supplementation to reduce the risk of blindness, infectious disease and mortality is high.

In 2008, WHO estimated that, since 1998 (when it and its partners started to deliver supplements through national immunisation days), 1.25 million VAD-related deaths had been prevented.

In 2008, the Copenhagen Consensus ranked the combined intervention of vitamin A and zinc supplementation as the world's best development investment. In 2010, the World Bank estimated that vitamin A supplements would have the highest cost-effectiveness of all mass nutrition interventions.

Increased awareness and availability of epidemiological information have enabled several countries to make sustained efforts to combat VAD through a combination of fortifying commonly consumed foods, providing supplements and, sometimes, dietary diversification.

Many countries have been successful in addressing VAD and are no longer considered to have a serious public health problem. In some countries, VAD has now virtually disappeared, for example in Guatemala and Nicaragua. This has been the result of combined interventions, including fortification, supplementation and home gardening.

**Figure:**
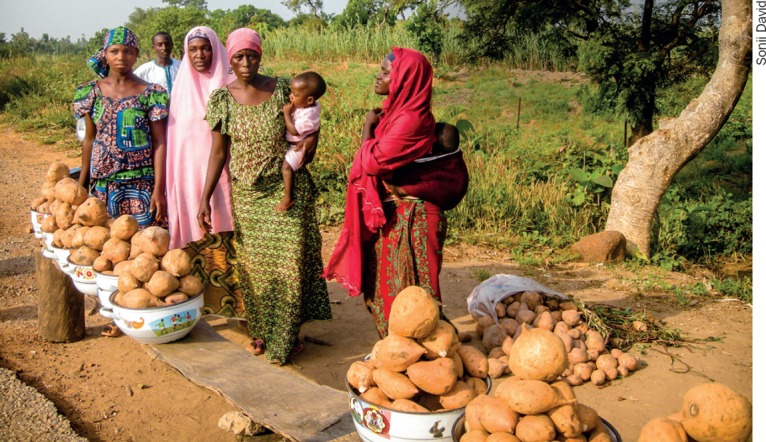
Women in Nigeria grow and sell orange-fleshed sweet potatoes, which are high in vitamin A

## Conclusion

The success of public health programmes for controlling VAD depend on the commitment, ownership and responsibility of governments, civil society and industry combined with advocacy and assistance from international agencies.

The ultimate aim should be that all children have a nutritious diet that includes foods rich in vitamin A. This can only be achieved through long-term development in agriculture and all the systems and processing required to ensure foods of high quality are available to all sectors of the population.

**Case study: Burkina Faso**

Burkina Faso initiated supplementation in 1986 after a survey showed that vitamin A deficiency was a major public health problem. Since then the country has used several different approaches.

1. Vitamin A supplementation has been integrated into national immunisation days, along with polio vaccination, in 1999; this improved coverage to over 90%. Since 2011, the country has held two ‘Vitamin A+ Days’ a year, during which supplements are given alongside other essential child survival interventions.
2. Increased consumption of vitamin A-rich foods is being promoted through school and community gardening programmes and nutrition education, with an emphasis on orange-fleshed sweet potatoes.
3. Dietary diversification is promoted through an enhanced homestead food production programme to improve year-round availability of a range of vitamin A-rich foods. Women learn to grow vitamin A-rich vegetable crops and raise chickens (for eggs) and goats (for milk).
4. Vitamin A-fortified foods are being produced through public-private partnerships with government ministries and commercial producers. Cooking oil produced in Burkina Faso is now fortified with vitamin A; 71% of the oil consumed in the country is now fortified with vitamin A.

The decline in child mortality from 184/1,000 to 129/1,000 during the last decade is one indication that these interventions have been effectively implemented.

A national survey to assess current levels of VAD will be conducted in 2014 in order to better inform vitamin A programme strategy.

*Written by Jean Celestine Somda, Laura Barrett, and Fanny Yago-Wienne* (*Helen Keller International, Burkina Faso*)*, and Heather Katcher and Jessica Blankenship* (*Helen Keller International, Africa Regional Office*).
